# Predicting functional outcome in bipolar patients: Effects of cognitive psychoeducational group therapy after 12 months

**DOI:** 10.1192/j.eurpsy.2021.244

**Published:** 2021-08-13

**Authors:** G. Sachs, A. Erfurth

**Affiliations:** 1 Department Of Psychiatry And Psychotherapy, Medical University of Vienna, Vienna, Austria; 2 1st Department Of Psychiatry And Psychotherapeutic Medicine, Klinik Hietzing, Vienna, Austria

**Keywords:** cognition, psychosocial functioning, cognitive psychoeducational group therapy, bipolar disorder

## Abstract

**Introduction:**

Cognitive impairment is known as a core feature in bipolar patients. Persisting neurocognitive impairment has been associated with low psychosocial functioning.

**Objectives:**

The goal of this work was to identify clinical and cognitive predictors for functional impairment, symptom severity and early recurrence in bipolar disorder, as well as to compare the neurocognitive performance of bipolar patients with that of healthy probands.

**Methods:**

43 remitted bipolar patients and 40 healthy controls were compared using a neurocognitive battery testing specifically attention, memory, verbal fluency and executive functions. In a randomized controlled trial, the 43 remitted patients were assigned to two treatment conditions as add-on to state-of-the-art pharmacotherapy: cognitive psychoeducational group therapy over 14 weeks or treatment-as-usual. At 12 months after therapy, functional impairment and severity of symptoms were assessed.

**Results:**

As compared to healthy probands, bipolar patients showed lower performance in executive function (perseverative errors p<0.01, categories correct p<0.001), sustained attention (total hits p<0.001), verbal learning (delayed recall p<0.001) and verbal fluency (pwords p<0.002). Cognitive psychoeducational group therapy and attention predicted occupational functioning with a hit ratio of 87.5%. Verbal memory recall was found to be a predictor for symptom severity (hit ratio 86.8%). Recurrence in the follow-up period was predicted by premorbid IQ and by years of education (hit ratio 77.8%).

**Conclusions:**

Our data show that bipolar patients benefit from cognitive psychoeducational group therapy in the domain of occupational life. Reductions in sustained attention have an impact on occupational impairment.

**Disclosure:**

No significant relationships.

